# Synergistic Effect of Enzyme Hydrolysis and Microwave Reactor Pretreatment as an Efficient Procedure for Gluten Content Reduction

**DOI:** 10.3390/foods10092214

**Published:** 2021-09-18

**Authors:** Ivana Gazikalović, Jelena Mijalković, Nataša Šekuljica, Sonja Jakovetić Tanasković, Aleksandra Đukić Vuković, Ljiljana Mojović, Zorica Knežević-Jugović

**Affiliations:** 1 Innovation Center, Faculty of Technology and Metallurgy, Karnegijeva 4, 11000 Belgrade, Serbia; igazikalovic@tmf.bg.ac.rs (I.G.); nsekuljica@tmf.bg.ac.rs (N.Š.); 2 Department of Biotechnology and Biochemical Engineering, Faculty of Technology and Metallurgy, University of Belgrade, Karnegijeva 4, 11000 Belgrade, Serbia; jjovanovic@tmf.bg.ac.rs (J.M.); sjakovetic@tmf.bg.ac.rs (S.J.T.); adjukic@tmf.bg.ac.rs (A.Đ.V.); lmojovic@tmf.bg.ac.rs (L.M.)

**Keywords:** wheat gluten, microwave reactor, allergenicity, enzymatic hydrolysis, Alcalase, techno-functional properties, antioxidant activity

## Abstract

In this study, we assessed the effects of microwave irradiation of wheat gluten proteins as a pretreatment performed in a microwave reactor that could accurately control process parameters as a function of power and temperature, as well as comparing it with conventional heat treatment. The aim was to identify suitable combinations of partial enzymatic hydrolysis and microwave pretreatment parameters to produce gluten hydrolysates with reduced allergenicity and conserved techno-functional features for food application. FTIR analysis, and total and reactive SH group contents confirmed that the microwave-controlled heating can significantly change the secondary structure and conformation of gluten protein. The microwave treatment had the largest effect at 200 W and 100 °C, at which the content of gluten has been reduced by about 2.5-fold. The microwave pretreatment also accelerated the enzymatic hydrolysis of gluten, changing the kinetic profile. The apparent hydrolysis rate constants (*k*_2_) were 1.00, 3.68, 3.48, 4.64 and 4.17 min^−1^ for untreated gluten, and those pretreated with microwave power of 200, 400, 600 and 800 W, respectively. Compared to the heat treatment, it appeared that microwave specific non-thermal effects had a significant influence on the gluten structure and allergenicity and, in combination with the enzymatic hydrolysis, ultimately yielded protein hydrolysates with enhanced antioxidant and functional properties.

## 1. Introduction

Apart from maize, wheat is the most globally cultivated cereal crop [1]. The unique properties of wheat flour, including specific viscoelastic properties, primarily reside in gluten-forming storage proteins of the wheat grain endosperm [2]. These unique features, together with the availability of wheat gluten as a by-product of the starch industry, have opened an enormous potential for a wide range of applications in the food bakery industry [3]. Although wheat gluten is the main determinant of dough properties, it also plays a vital role in the production and/or quality attributes of other foods by modification of the rheological, textural, and organoleptic properties of the final products. Moreover, it is possible to enlarge the field of gluten applications through its chemical or enzymatic modifications, with the aim of improving functional and nutritional properties for specific food and other applications [4,5,6]. 

Gluten makes up 80–85% of the total protein content in wheat, while 15–20% of the remaining proteins are non-glutenous [7]. Protein bodies found in the wheat endosperm contain prolamins, which represent the major gluten fraction, and other gluten constituents [8]. Wheat gluten is a rather complex protein composed of two seed storage proteins, gliadins and glutenins [9]. Glutenins, the major proteins of flour, are poorly soluble in alcohol because they are capable of forming large polymers that are stabilized by intermolecular disulfide bonds and hydrophobic interactions. On the other hand, gliadins are soluble in aqueous alcohol (for example 60–70% ethanol) and are mainly present in gluten as monomers interacting by non-covalent forces [10,11]. However, wheat storage protein, including gliadin and glutenin, are more complex proteins which are comprised of polymorphic polypeptides, showing more than 60 different molecular weights ranging from 30,000 to 90,000 Da. They can be closely divided into several groups according to their molecular weight (MW): (1) high molecular weight (HMW) group—HMW-glutenin subunits (HMW-GS), x- and y-type, with MW ranging 70–90 kDa; (2) medium molecular weight group (MMW)—ω5- and ω1,2-gliadin, with MW ranging 40–50 kDa; (3) low molecular weight (LMW) group—*α*/*β*- and γ- gliadin occurring as monomers, LMW-glutenin subunits (LMW-GS) occurring as aggregative proteins, with MW ranging 30–40 kDa [12]. Depending on the MW group, the proteins have different amino acid compositions [12,13].

Gluten proteins are generally rich in proline residues, making them indigestible and thus trigger an immune response in predisposed individuals [14]. Celiac Disease (CD) refers to chronic digestive problems and nutritional deficiencies. It is defined as an inflammatory disease of the upper small intestine and is caused by the consumption of gluten-containing foodstuff. The disease is chronic, and the only effective treatment is a strict, lifelong elimination of foods containing gluten from the diet, which is a big challenge for CD patients due to the frequent usage of gluten in the food industry [15,16]. Thus, food science research has paid considerable attention to the development of processing technology that reduces or eliminates “toxic” gluten and other protein sequences in raw materials and foods [17].

Enzymatic treatment of gluten seems to be a highly promising approach, aiming at the hydrolysis of toxic gluten sequences in vitro prior to ingestion; it has also been suggested as as an oral therapy for CD, in which dietary gluten is hydrolyzed by digestive peptidases which are already in the stomach, thus preventing CD-specific immune reactions in the small intestine (so-called medical approach) [18]. Even though oral therapy for celiac disease by digestive peptidases is an attractive approach, it creates several technological challenges such as ensuring rapid and complete enzymatic digestion of immunogenic gluten peptides in complex food matrices. Furthermore, some strongly allergenic sequences of the 33-mer peptide, from α/β-gliadins, seem to be resistant to gastrointestinal digestion [13]. Recent research has confirmed the potential of prolyl endopeptidases from different sources alone or in combination with cysteine endoprotease to detoxify gliadin peptides, but raise concerns regarding their possible efficacy in vivo, in the intestinal environment and in CD [13,19,20]. Namely, the ability of the enzyme to diffuse and access the epitopes is reduced by food matrix components, leading to incomplete allergen destruction. Additionally, the combination of low pH and the presence of the pepsin in vivo could enhance the inactivation of the enzyme, thus high concentrations and long reaction times are required to achieve complete detoxification and to prevent intestinal transport of toxic sequences [13,21]. Engineered synthetic and improved gluten-degrading enzymes, their combination and microencapsulation appear to be a future direction in enzyme therapy, but they are still in progress and in various phases of clinical testing [22,23,24,25]. On the other hand, enzymes with broader specificity such as Alcalase may hydrolyze in vitro more peptide bonds under selected reaction conditions and expose new sites that may not have been available to more specific enzymes [26]. The use of enzyme for in vitro gluten hydrolysis can be particularly advantageous since the enzymes are able not only to eliminate contaminant gluten, but also to enhance solubility and to obtain the required nutritional and functional properties [13,27]. Moreover, the hydrolysis may also produce bioactive peptides with various functionalities and potentially biological activities.

To the best of our knowledge, the enzymes obtained from various sources have been used for modifying the immunogenic fraction of gluten proteins. It appears to cause several changes in gluten’s structure by cutting the large proteins to peptides of lower molecular weight, reducing the allergenic potential of wheat gluten since its allergenic epitopes contain between 5 and 20 amino acids [28]. However, the development of an efficient enzymatic processing technology for industrial application still requires progress. It is important to completely eliminate residual allergenic epitopes since they could have a deleterious effect on product quality and the health of coeliac patients during processing and subsequent peptic and tryptic digestion. Furthermore, due to the presence of large amounts of hydrophobic residues in hydrolyzed gluten fraction, the proteins typically have poor solubility and dispersibility. For example, the modified gluten network after endopeptidase treatment reduces the technological properties (viscoelasticity) of dough and baked products, which are supplemented by structuring agents such as pre-gelatinized starch, emulsifiers, and hydrocolloids [29,30]. This is the main disadvantage of the enzyme treatment. An effective treatment method must be able to cleave or mask the amino acid sequence present in toxic epitopes at a specific position or to alter the protein conformation of the allergen (protein denaturation, cross-linking, or aggregation). During the last decade, various approaches which imply the combination of partial and controlled enzyme hydrolysis with some physical pretreatment of gluten proteins and its fractions were applied and gave the possibility for obtaining final products with conserved techno-functional features [30,31,32].

The aim of this research was to utilize the advantages of a fully controlled and equipped microwave reactor system as a pretreatment step in order to facilitate the enzymatic hydrolysis reaction and to investigate the changes in wheat gluten proteins which occur during microwave treatment (MWT). This was conducted in order to investigate the MWT effects on gluten molecular structure and the possibility of certain toxic epitopes to become more easily available to the enzyme, which may favor their elimination. Conventional heating was also applied in order to compare its effects to the effects of microwave energy regarding gluten allergenicity. Changes in functional, antioxidant and metal-chelating properties were also investigated.

## 2. Materials and Methods

### 2.1. Materials

Gluten from wheat (min. 75% protein content, as declared by the supplier) and Alcalase 2.4L, a serine endopeptidase from *Bacillus licheniformis*, were obtained from Sigma-Aldrich (St. Louis, MO, USA). Bovine serum albumin (BSA), 2,2′-Azino-bis (3-ethylbenzothiazoline-6-sulfonic acid) diammonium salt (ABTS), 3-(2-Pyridyl)-5,6-diphenyl-1,2,4-triazine-4′,4′′-disulfonic acid sodium salt (ferrozine), 5,5′-(dithiobis-2-nitrobenzoate) (DTNB), FeCl_2_ were also procured from Sigma-Aldrich (St. Louis, MO, USA). RIDASCREEN^®^ Gliadin competitive (Art. No. R7021) ELISA test (R-Biopharm AG, Darmstadt, Germany) was used to quantify gliadin reactive epitopes. All of the other chemicals were of analytical grade.

### 2.2. Methods

#### 2.2.1. Microwave Reactor Treatment

Microwave treatment was conducted using the microwave reactor system Anton Paar Monowave 300 (Anton Paar GmbH, Graz, Austria). A sample of 1 g of wheat gluten was weighed into designated reactor vials (G30) and distilled water was added, the vials were capped to avoid evaporation and then the samples were subjected to treatment. Such formed 10% (*w*/*v*) suspensions were treated by different constant microwave power, ranging from 200–800 W with magnetic stirring fixed to 600 rpm. Reaction temperature maximum was set at 100 °C and different microwave powers were applied. Treatment time at 100 °C was 1 min. Treated samples were then lyophilized (CHRIST Beta 2-8 LDPlus, Osterode am Harz, Germany) and stored in tightly secured plastic vials in a glass desiccator at room temperature for further analysis. The microwave power chosen for further experiments was based on the immunoassay results. Afterwards, the chosen microwave power was used in a set of experiments where the temperature was controlled and varied from 50–100 °C with an increment of 10 °C. Magnetic stirring speed was held constant and treatment time was 1 min. Total protein content (*N* × *5.7*) was determined by the Kjeldahl method [33]. The experiments were performed at room temperature (~25 °C).

#### 2.2.2. Conventional Heat Treatment

Heat treatment was conducted using a heating unit with temperature control (Heidolph MR 3001, Heidolph Instruments GmbH & Co. KG, Schwabach, Germany) with an oil bath. The 10% (*w*/*v*) wheat gluten suspensions were heated to 50–100 °C range, with an increment of 10 °C, with magnetic stirring set to 600 rpm and samples were treated for 1 min at the designated temperatures. Samples were lyophilized (CHRIST Beta 2-8 LDPlus, Osterode am Harz, Germany) and stored in tightly secured plastic vials in a glass desiccator at room temperature for further analysis.

#### 2.2.3. Enzymatic Hydrolysis

Immediately after the microwave reactor treatment, the sample was subjected to hydrolysis in a stirred tank reactor which consisted of pH electrode (Eutech instruments Pte Ltd, Singapore), overhead stirrer (Heidolph RZR 2020, Heidolph Instruments GmbH & Co. KG, Schwabach, Germany) and a heating unit (IKA^®^ C-MAG HS 7, IKA^®^–Werke GmbH & Co. KG, Staufen, Germany), where the agitation speed was set to 200 rpm. The 2% (*w*/*w*) wheat gluten suspensions (total mass of 200 g in a 400 mL beaker) were stirred and allowed to equilibrate at 60 °C for 20 min. The reaction pH was adjusted to pH 8.0 using 0.8 N NaOH. The hydrolysis reaction was started by addition of Alcalase, where (E/S) ratio was 5.0%. Enzymatic hydrolysis was monitored by pH-stat assay, where DH was calculated according to Adler-Nissen [34], as shown in Equation (1):

(1) $$DH\left(\%\right)=\frac{\left(h\times 100\right)}{h_{\mathrm{tot}}}=\frac{N_{b}\times B\times 100}{\alpha \times m_{p}\times h_{\mathrm{tot}}}$$

where *h* is the number of equivalent peptide bonds hydrolyzed at the time expressed in meq/g, *h*_tot_ is the total amount of peptide bonds per weight unit of a protein (for wheat gluten protein *h*_tot_ = 8.38 mmol/g of protein), *N*_b_ is the base normality, *B* is the amount of base consumed in mL, *α* is the degree of dissociation of *α*-amino groups (*α* = 0.926 at 60 °C and pH 8.0) and *m*_p_ is the protein mass in g. The hydrolysis was terminated by boiling the samples for 10 min and centrifuged at 7860× *g* at 4 °C (Eppendorf Centrifuge 5430 R, Hamburg, Germany). The samples were lyophilized (CHRIST Beta 2-8 LDPlus, Osterode am Harz, Germany) and stored in tightly secured plastic vials in a glass desiccator at room temperature for further analysis. Protein content in the hydrolysates was determined by the method of Lowry [35].

In order to interpret and better understand the obtained experimental results, a semi-empiric kinetic model that takes into account the enzyme deactivation and substrate inhibition was applied [36]. Afterwards, the kinetic parameters were calculated, and the initial rate of gluten proteins hydrolysis was interpreted via reaction rate constant (*k*_2_), inhibition constant (*K*_I_) and reaction rate constant of deactivation (*k*_d_).

#### 2.2.4. Quantification of Gliadin Reactive Epitopes

The gliadin content was determined in the untreated wheat gluten, microwave reactor treated gluten, conventionally heated gluten and in-wheat gluten hydrolysates with and without microwave reactor pre-treatment by using the ELISA RIDASCREEN^®^ Gliadin Competitive kit (R-Biopharm, Darmstadt, Germany). The assay was performed according to the manufacturer’s instructions.

#### 2.2.5. Emulsifying Activity and Emulsion Stability Index

Emulsifying activity index (*EAI*) and emulsion stability index (*ESI*) were determined by the method of Pearce and Kinsella [37], with modifications. All of the previously lyophilized samples were dissolved in distilled water at 1.0% (*w*/*v*). The working solution consisted of 3/4 dissolved sample and 1/4 of sunflower oil and was mixed for 90 s using a laboratory homogenizer (Yellowline DI 25 basic, IKA^®^ Works, Inc., Wilmington, NC, USA) at a speed of 9500 rpm. An aliquot of 50 μL was diluted 100 times with phosphate buffer (0.01 M, pH 7) containing 0.1% (*w*/*v*) SDS. The absorbance of the diluted emulsions was measured at 500 nm at 0 min (*A*_0_) and at 10 min (*A*_10_) at ambient temperature. The calculations are given in Equations (2)–(4):

(2) $$T=\frac{2.303\times A}{l}$$

(3) $$EAI\left(\frac{m^{2}}{g}\right)=\frac{2\times D\times T}{\varphi \times c\times 10,000}$$

(4) $$ESI\left(h\right)=\frac{\left(A_{0}\times \Delta t\right)}{\Delta A}$$

where *T* is turbidity, *A* is the absorbance measured at 500 nm (at 0 and 10 min), *l* is the optical path length of cuvette = 1 cm, *D* is the dilution factor = 100, *c* is the weight of protein per unit volume (g/mL), *φ* is the oil volume fraction in the emulsion, ∆*A = A_0_ − A_10_* and ∆*t* = 10 min is the time interval.

#### 2.2.6. Foam Capacity and Foam Stability

All of the samples were dissolved to form a 2.0% (*w*/*v*) solution. The method was slightly modified [38]. The initial volume of 50 mL of each sample solution was recorded and then the solution was whipped for 4 min with a laboratory homogenizer at 9500 rpm at ambient temperature. The same beaker was used for all samples. Immediately after whipping, the beaker was sealed with parafilm in order to evade air contact. Foam capacity (*FC*) was calculated as foam expansion at 0 min, as given in Equation (5):

(5) $$FC\left(\%\right)=\frac{V_{A}-V_{B}}{V_{A}}\times 100$$

where *V*_A_ is the volume after whipping (mL) and *V*_B_ is the volume recorded before 4 min of whipping (mL). 

Foam stability (*FS*) was calculated as the percentage of liquid present in the foam after 30 min compared to the solution recorded at 4 min after whipping. The calculation is given in the Equation (6):

(6) $$FS\left(\%\right)=\frac{V_{A}-V_{B}}{V_{A}}\times 100$$

where *V*_A_ is the volume recorded after 30 min of rest (mL) and *V*_B_ is the volume recorded before 4 min of whipping (mL).

#### 2.2.7. ABTS˙^+^ Radical Scavenging Activity

The antioxidant properties of untreated wheat gluten, microwave reactor treated gluten, conventionally heated gluten, and wheat gluten hydrolysates with and without microwave reactor pretreatment were determined by a free-radical scavenging assay, ABTS, with modifications as described [39]. Determination of the ability of the aforementioned samples to scavenge the ABTS˙^+^ radical was based on the ABTS˙^+^ radical cation decolorization. The samples were prepared as 2 mg_protein_/mL solutions in distilled water and vortexed. Then, 10 μL of prepared solutions were mixed with 1 mL of previously prepared ABTS˙^+^ solution. The incubation time lasted for 5 min and absorbance was measured at 734 nm. ABTS radical scavenging activity (%) was calculated using Equation (7):

(7) $$ABTS\left(\%\right)=\left(1-\frac{A_{S}}{A_{C}}\right)\times 100$$

where *A*_s_ represents the absorbance of the sample solution in the presence of ABTS˙^+^ and *A*_c_ is the absorbance of the control solution with ABTS˙^+^. Minimal inhibitory concentration of hydrolysates necessary to inhibit 50% of ABTS radical cation, at standard reaction conditions, was calculated and expressed in mg/mL.

#### 2.2.8. Metal-Ion Chelating Activity

The metal-ion chelating activity (MICA) of the wheat gluten hydrolysates was determined using a ferrous ion chelating assay described by Decker and Welch [40], with modifications. A sample of 200 μL of each hydrolysate solution (4 mg_protein_/mL in deionized water) was added to 800 μL of deionized water. Then, 100 μL of 2 mM FeCl_2_ solution was added, vortexed and incubated for 3 min. Afterwards, 200 μL of 5 mM ferrozine solution was added, vortexed and incubated for another 10 min. The absorbance was recorded spectrophotometrically at 562 nm, with deionized water as blank. Metal-ion chelating activity was then calculated as given in Equation (8):

(8) $$MICA\left(\%\right)=\left(1-\frac{A_{1}}{A_{0}}\right)$$

where *A*_0_ is the absorbance of the control and *A*_1_ is the absorbance of the sample, both measured at 562 nm. Minimal inhibitory concentration of hydrolysates necessary to chelate 50% of ferrous ions, at standard reaction conditions, was calculated and expressed in mg/mL.

#### 2.2.9. Quantification of Total and Reactive SH Groups

For the purpose of verifying the influence of microwave pretreatment on the potential structural changes of gluten proteins, the effect of microwave heating and conventional heat pretreatments on the changes in content of total sulfhydryl and reactive (SH) groups was determined spectrophotometrically by Ellman’s procedure using 5,5′-(dithiobis-2-nitrobenzoate), DTNB, which reacts with exposed SH groups to yield a product with a maximum absorbance at 412 nm. Analysis was conducted as previously described [41,42].

#### 2.2.10. FTIR Analysis

Fourier transformation infrared spectroscopy (FTIR) absorbance spectra of lyophilized samples were acquired using Nicolet iS10 FTIR Spectrometer (Thermo Scientific™). Absorbance spectra at 4 cm^−1^ resolution were collected over the scanning range of 400 to 4000 cm^−1^. The background of spectra was corrected by spectrum of air. All of the analyses were performed at room temperature.

#### 2.2.11. Sodium Dodecyl-Sulfate Polyacrylamide Gel Electrophoresis (SDS-PAGE) Electrophoresis

SDS-PAGE electrophoresis was performed on hydrolysate samples using a 12% Precise™ Protein Gel on a Hoefer™ Mighty Small™ II Mini Vertical Electrophoresis System. Hydrolysate samples and sample buffer were mixed in a 1:1 ratio and boiled for 5 min. Afterwards, 20 μL of the reduced protein sample was used to load on to the separating gel. The separation was performed under 40 mA current for 100 min. Spectra Multicolor Broad Range Protein Ladder (Thermo Scientific), a protein standard containing 10 pre-stained proteins with molecular weights ranging 10–260 kDa, was used. The gel was stained with Coomassie Brilliant Blue R-250. Molecular weights (Mw) were then estimated on the basis of the protein standard.

#### 2.2.12. Statistical Analysis

Two independent experiments were performed for each of the experimental sets (microwave or heat treatment; enzymatic hydrolysis), and the results are presented as the mean values with standard deviations (SD). One-way ANOVA with repeated measures (within subjects), followed by Tukey’s test, was used to determine the statistical significance of mean values differences for comparison of gluten treatments at level less than 0.05. On the contrary, one-way ANOVA followed by Tukey’s test was used to examine the relationship between the studied parameters, i.e., dependent variables of two and three technical repeats per allergenicity detection and antioxidant and functional analyses, respectively (significance level was *p* < 0.05). Statistical analyses were performed using OriginPro 9.0 software (OriginLab Corporation, Northampton, MA, USA).

## 3. Results and Discussion

### 3.1. Microwave-Enhanced Heating Process Induced Changes in Gluten Allergenicity Detection

**Figure 1:**
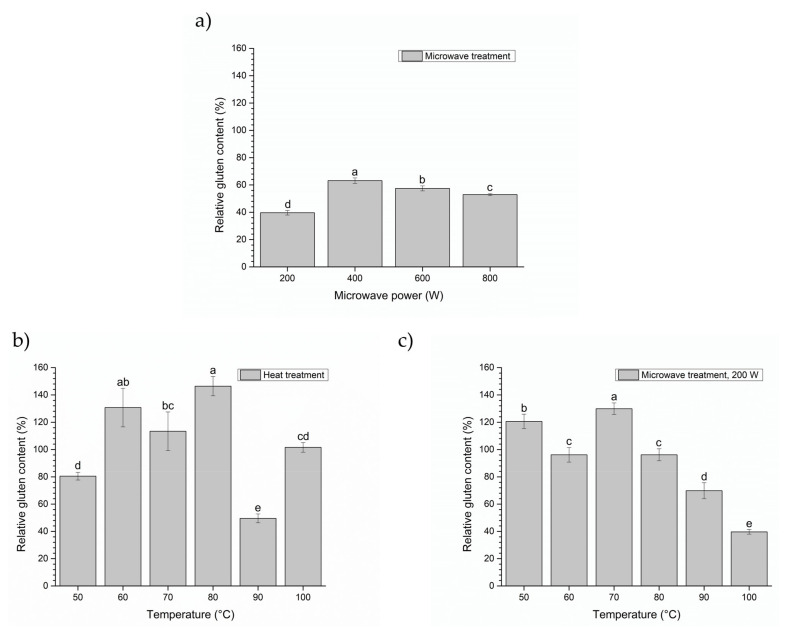
Relative gluten content (%) detected after (**a**) microwave treatment of wheat gluten at different microwave powers (200–800 W); (**b**) heat treatment of wheat gluten at different temperatures (50–100 °C); and (**c**) microwave treatment of wheat gluten at 200 W at different controlled temperatures (50–100 °C). All measurements were compared to an untreated gluten sample, considered as control (100%). Results are expressed as mean ± standard deviation (*n* = 2). Means with different letters in the same figure are significantly different (*p* < 0.05).

The effects of microwave irradiation on natural gluten proteins as a pretreatment performed in a microwave reactor were studied as a function of reaction parameters, power and temperature, and compared with conventional heat treatment. Since wheat gluten as a raw natural material contains impurities, including carbohydrates and proteins which are very sensitive to temperatures above 90 °C due to denaturation and degradation of natural compounds as well as adverse reactions such as Maillard reaction and others, the tendency to find alternative less aggressive physical treatments is increasingly pronounced [43]. The aim is to obtain modified gluten proteins in high yield with a lower value of toxic immunogenic epitopes and preserved techno-functional properties, which further implies that special conditions are necessary to perform the reaction process. Thus, the first step was focused on the investigation of the possibility to apply a physical treatment on the gluten proteins by using a microwave reactor with temperature control in order to modify gluten molecules in a way which will be able to influence the reduction in gluten allergenicity. The influence of the microwave irradiation power (200–800 W) and the temperature (50–100 °C) under controlled conditions was examined, and the obtained results were presented in Figure 1.

The results revealed that the microwave treatment of wheat gluten resulted in gluten content reduction in all of the treated samples (Figure 1a) in comparison with untreated gluten. The greatest reduction in detected relative gluten content was recorded for the sample treated at power of 200 W, retaining 39.65 ± 1.69% of its initial gluten content. At low power, an initial decrease in content of toxic gluten epitopes appeared, then the lowest reduction in toxic gluten epitopes was attained at 400 W (63.16 ± 1.98% of initial content) and, at that point, the decrease in relative gluten content at higher applied power was observed. The mechanism of the microwave influence on gluten allergenicity at the molecular level appeared to be rather complex and based on the alteration of the protein conformation of epitopes. It appeared that the denaturation and unfolding of the gluten molecule were higher at 400 W than at 200 W, thus rendering hidden epitopes more accessible by the antibody used in ELISA, resulting in higher antigenicity. However, further increasing of microwave power to 800 W could have a different effect on gluten structure including disulfide bonded cross-linking between gliadin and glutenin, which could cause destruction and/or masking of some epitopes, or additional denaturation of epitope resulting in reduced antigenicity. Furthermore, the aggregation and loss of protein solubility, rather than the epitope destruction, may be responsible for the observed decrease in gluten immunoreactivity at higher microwave power. Thus, future studies on immunoreactivity of different soluble and insoluble gluten fractions are required to additional understand the mechanisms of the inactivation of gluten toxic epitopes by microwave treatment [44]. Similar studies have demonstrated that at lower dose of applied energy, the gliadin immunoreactivity increased, reached the maximum value; at higher applied doses (500 W for 2 min), a decrease in gliadin immune response was observed [45]. Based on the results obtained, the microwave power of 200 W was selected to examine the effect of temperature on the detectable value of gluten content.

In order to compare microwave treatment to the effect of conventional heat treatment, gluten samples were treated on a heating unit to 50–100 °C with an increment of 10 °C, under the same conditions. The same temperatures were set on the microwave reactor and power of 200 W was applied. The results (Figure 1b,c) showed that gluten samples treated with microwave power, exhibited somewhat of a declining trend in relative gluten content value for the selected temperatures. The lowest relative gluten content value detected for conventionally heated gluten samples was at 90 °C and was 49.56 ± 3.25%, while the microwave treatment was most effective for the detoxification of gluten at the highest temperature (100 °C), at which the relative gluten content was 39.65 ± 1.69%. These results suggested that the gluten allergenicity detection was greatly affected by microwave treatment. Namely, an approximate 2.5-fold reduction in detected gluten content was achieved through the simple application of 200 W of microwave power. As microwave heating and conventional heating at the same temperature showed rather different effects on gluten content, it can be concluded that microwave specific effects (non-thermal effects) had influence on the gluten structure and on the gluten allergenicity. Thus, it can be emphasized that the microwave treatment under controlled conditions destroys the network of hydrogen bonds, causing changes in dipole rotation of gluten protein molecules and migration of ions in the working aqueous environment [46].

Compared to the heat treatment, the application of microwave treatment is a promising alternative since the reaction time is drastically reduced, which increases the economy of the treatment process and the exposure of gluten proteins to temperatures for a shorter period of time, which affects their characteristics. The time required for the microwave treatment of 200 W to reach the 50–100 °C temperatures was a few seconds, compared to heat treatment where 50–100 °C were reached in 2.75–25 min. Furthermore, the heat treatment appears to be undesirable because of its known negative effects on protein nutritional and functional properties as a consequence of the cross-linking of disulfide peptide bonds that take place by the mechanism of acylation of free amino groups [47]. The amino acid lysine is the most susceptible to this type of reaction, but also the amino acids serine, cysteine and cystine, as well as tryptophan-giving reaction products that significantly reduce the nutritional value of proteins. Generally speaking, the process involves the application of high pressures, shear force, as well as high temperatures for a long period of time result in irreversible denaturation of gluten proteins caused by deamidation of aspartic acid and glycine residues, disruption of peptide bond on aspartic acid, and destruction of amino acid residues [48]. Similarly, Lamacchia et al. [46] reported that the gliadins from treated flours (microwave oven: 1000 W, 2 min of treatment to reach a temperature of 110–120 °C) showed significantly reduced cross-reactivity with the R5 antibody. However, although the microwave pretreatment has been found to lead to gluten secondary structure alterations related to the polymer’s disaggregation phenomenon, the pretreatment inefficiency to detoxify the gluten for celiac disease patients was observed [44].

There are many contradictory reports on the influence of microwaves on gluten celiac-related toxicity and there is scientific proof indicating inefficiency of this approach [44,49]. For example, although the microwave treatment of soaked wheat kernels has been documented to decrease the toxic epitope content in gluten with the R5-ELISA assay and also, after deamidation, with in vitro assay on gut-derived T-cell lines of celiac patients [48,50], other works reported that it neither destroyed the gluten nor chemically modified the toxic epitopes [44,49]. Namely, a recent paper by Gianfrani et al. [49] demonstrated that, by LC-MS/MS and by in vitro assay with T-cells of celiac patients, despite the early encouraging results about the drastic reduction in R5-immunoreactivity of the undigested soluble microwave treated gluten fraction, the MWT did not affect immune toxicity of gluten [49]. They confirmed that the treatment altered the wheat kernel protein solubility and apparently caused a drastic reduction in gluten, up to 70 ppm, which is in accordance with a previous report [48], but also established that the immunoreactive R5 gliadin components of microwave treated kernels were not simply extracted, most likely remaining attached to the substrate due to protein denaturation. Thus, the differences in the literature may be explained by different gluten extraction procedures and analytical tools for gluten detection, as well as different operating systems of the microwaves which mostly have not been specified. Moreover, in order to reproduce the same temperature and energy profile in the sample, time and power control are very important in this process. The present results confirm the potential of the designed MWT to detoxify wheat gluten but raise concerns regarding its possible efficacy in vivo, in the intestinal environment. Thus, the aim of the present research is to overcome the disadvantages of prior investigations by proposing a method of detoxification whereby gluten is based on the combination of microwave treatment and enzymatic hydrolysis to achieve better effects.

### 3.2. Combined Effect of Microwave Pretreatment (MWT) and Biocatalyst Alcalase on Gluten Content Reduction

The next series of experiments were performed in order to investigate the combined effect of the MWT and biocatalyst Alcalase on the detectable gluten content in the resulting hydrolysates (MWGHs). The starting point for this study was the finding that the microwave treatment alone resulted in the retention of toxicity and, on the other hand, the high proline content in gluten makes it highly resistant to complete enzymatic hydrolysis.

**Figure 2:**
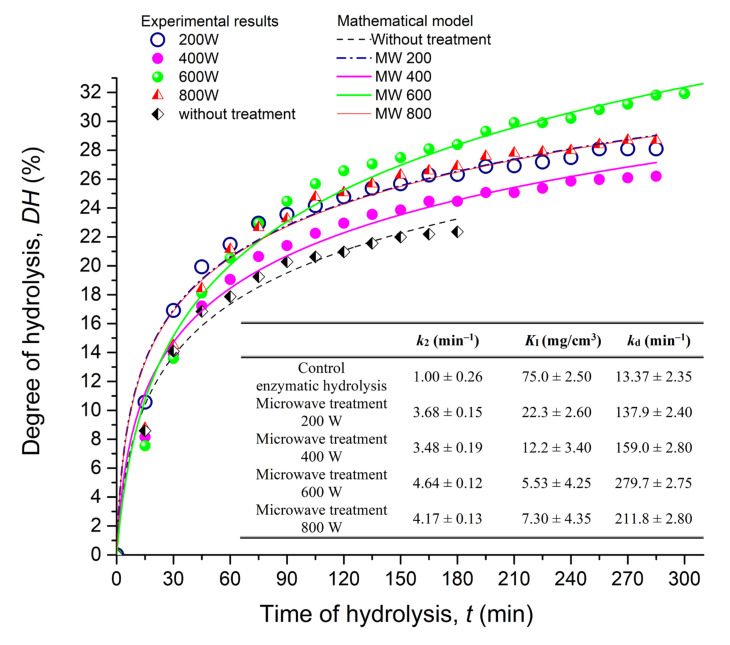
Comparison of the susceptibility of differently microwave treated gluten proteins to enzymatic hydrolysis conducted with commercial food-grade protease, Alcalase. The experimental results were fitted by using the empirical kinetic model with substrate inhibition and enzyme deactivation. Reaction conditions for hydrolysis of presented curves: gluten concentration 2% (*w*/*w*), *E*/*S* ratio 5%, temperature 60 °C and pH 8). Inserted table—Values of the kinetic constants for enzymatic hydrolysis of microwave pretreated gluten proteins.

In order to investigate the combined effect of microwave pretreatment and a biocatalytic process on gluten content detection, microwave reactor powers of 200, 400, 600 and 800 W were applied. The pre-treatment step was followed by immediate gluten hydrolysis with Alcalase (Figure 2) and the aim of this experimental setup was to investigate whether microwave energy can facilitate the hydrolysis of toxic epitopes. By using a microwave reactor Anton Paar Monowave 300, it is possible to completely control the treatment conditions: temperature, treatment time and power, which is of great importance when it comes to protein treatment, where the pretreatment process itself can directly affect the final protein characteristics.

It is obvious from Figure 2 that the microwave-enhanced heat treatment significantly changed the hydrolysis pattern of gluten proteins (*p* < 0.05) in all of the examined irradiation powers (200–800 W). The initial reaction rate increased with the increase in the microwave power, while the achieved degree of hydrolysis increased with the increase in power up to 600 W, followed by a decrease when 800 W was applied. The achieved degree of hydrolysis varied from 20% to 32% over the 300 min time period. The hydrolysis proceeded at a rapid rate during the initial 45 min of the reaction and the recorded *DH* was about 16% for CGH, and more than 17% for MWGHs. Afterward, the enzymatic hydrolysis proceeded with a slow increase in hydrolysis rate, for the next 150 to 200 min, and then entered a steady state. In this way, it was confirmed that the microwave-treated gluten proteins as substrate had a considerable susceptibility to Alcalase; thus, the prepared hydrolysates could be used to identify their allergenicity characteristics. The represented *DH* profile with time is similar to the typical hydrolysis curve reported by Elmalimadi et al., (2017) [27] and Kong et al., (2007) [26]. The evident initial increase in susceptibility to enzymatic hydrolysis may be attributed to microwave-heat-induced conformational changes of gluten molecules, which can cause full or partial unfolding of polypeptides and resulted in exposure of buried peptide bonds, making them more accessible to the enzyme attack. However, treatment at 800 W caused a significant decrease (*p* < 0.05) in the susceptibility to enzymatic hydrolysis compared to 200 W, suggesting the previously mentioned gluten aggregation which in turn protected the internal bonds of the proteins.

To quantitatively compare the effects of microwave treatment power, the experimental data were fitted to a semi-empiric kinetic model that took into account the enzyme deactivation and substrate inhibition. The predicted mathematical kinetic model, considering the hydrolysis reaction as a zero-order reaction, aligned well with the obtained results, *R*^2^ ≥ 0.98. Based on the calculated values for the apparent reaction rate constant (*k*_2_), the microwave pretreatment enhanced the reaction rate of enzymatic hydrolysis of gluten when compared to the gluten without treatment (Table inserted in Figure 2). For microwave treatment at power of 200 W, a 3.6-fold increase in the reaction rate constant appeared, but the overall level of gluten proteins hydrolysis, *DH* increased only 3%. However, at more intensive microwave treatment at a power of 600 W, the 4.6-fold increase in reaction rate constant led to a significant enhancement of *DH*, even ~9%. Both inhibition and reaction rate constants of deactivation also varied significantly along the microwave power, ranging from 5.53 to 22.3 mg/cm^3^ and 137.9 to 211.8 min^−1^, respectively. It is plausible that the protein microwave pretreatment caused the structural changes of gluten molecules to a form having an increased susceptibility to Alcalase but decreased binding to enzyme that may affect both enzyme inhibition by substrate (*K*_i_) and enzyme stability (*k*_d_).

**Figure 3:**
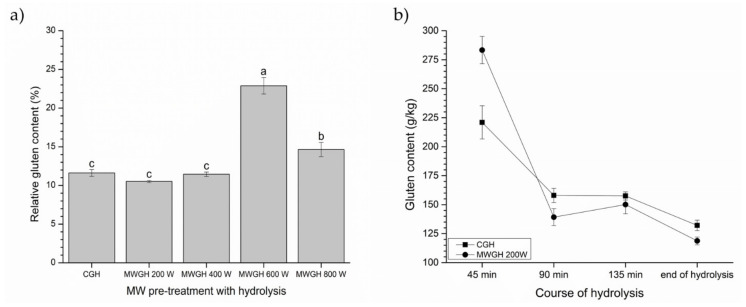
(**a**) Relative gluten content (%) of the control gluten hydrolysis (CGH) and microwave pretreated gluten hydrolysates (200–800 W) (Alcalase, pH 8, 60 °C) compared to untreated gluten (100%) and (**b**) gluten content reduction (g/kg) during hydrolysis, CGH and MWGH 200 W. Results are expressed as mean ± standard deviation (*n* = 2). Means with different letters in the same figure are significantly different (*p* < 0.05).

The application of 600 and 800 W did not result in a significant gluten reduction (*p* > 0.05, Figure 3a). However, the relative gluten content of CGH and MWGH samples pretreated at 200 W and 400 W significantly improved compared to untreated gluten; samples showed an approximate 10-fold reduction. Nevertheless, the pretreatment at 200 W has proven to be the most effective; therefore, a closer comparison with the control hydrolysis was investigated (Figure 3b). In order to investigate whether a complete enzymatic hydrolysis needed to be performed in order to achieve the greatest gluten content reduction, samples were taken at different time of hydrolysis, lyophilized and gluten content was determined. It was apparent that the treatment at 200 W slightly contributed to reducing gluten content of the hydrolysate compared to CGH. The treatment had the highest contribution after 90 min when the content of gluten was reduced by an estimated 11.86%. It was apparent that the prolonged reaction time above 90 min did not support significantly towards further detoxification of gluten.

### 3.3. Influence of Microwave Pretreatment on Structural Changes of Gluten Proteins

**Figure 4:**
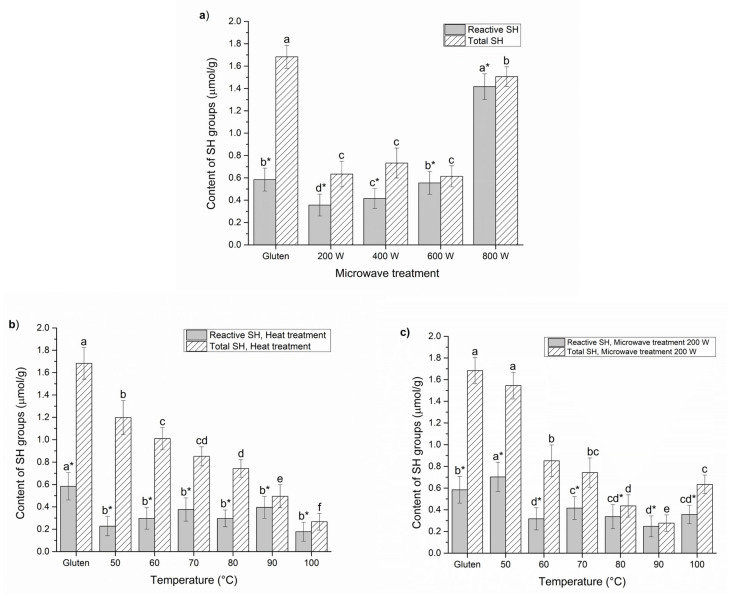
Content of total and reactive SH groups detected after: (**a**) microwave treatment of wheat gluten at different powers (200–800 W); (**b**) heat treatment of wheat gluten at different temperatures (50–100 °C); and (**c**) microwave treatment of wheat gluten at 200 W and controlled different temperatures (50–100 °C). All measurements were compared at the same protein concentration 2 mg/mL. Results are expressed as mean ± standard deviation (n = 3). Means with different letters in the same figure are significantly different (*p* < 0.05).

In an effort to better understand the previous results and the influence of microwave pretreatment on the enzymatic hydrolysis, its impact on the structure of gluten main protein fractions in terms of total and reactive SH group contents and FTIR analysis were performed and compared with conventional heat treatment. The obtained results are presented in Figure 4.

It seemed clear that both content of reactive and total SH groups were strongly dependent on microwave power and temperature, but also the type of treatment. Regarding the content of SH groups presented in Figure 4a, the microwave treatment of gluten protein resulted in a statistically significant (*p* < 0.05) increase in the content of reactive SH groups, at the power of 800 W, which showed an advantage over the lower microwave powers. Control gluten sample demonstrated significant differences between the amount of total and reactive SH groups, 1.68 ± 0.103 and 0.58 ± 0.103 µmol/g, respectively. The increase in the content of total and reactive SH groups at 800 W could be explained by heat-induced conformational changes, which can cause partial unfolding of polypeptides, resulting in exposure of buried SH groups (reactive SH = 0.36 to 1.40 µmol/g and total SH = 0.63 to 1.50 µmol/g). Namely, during microwave treatment, most SH residues in gluten that existed in the interior of protein molecules were exposed with controlled-heat denaturation. As the susceptibility of gluten to hydrolysis after MWT 600 W was rather high (*DH* ~ 32%), the decrease in total SH content might be explained by the formation of predominantly intramolecular disulfide bonds due to sulfhydryl oxidation. On the other side, a MWT at a power greater than 600 W, the increase in total SH groups may attribute to disruption of intramolecular disulfide bonds induced by a temperature higher than 100 °C. Besides, decrease in protein susceptibility to hydrolysis and increase in substrate inhibition could be associated with the presence of intermolecular disulfide bond, leading to the protein aggregates formation. The protein aggregates formation is in accordance with the measurement of detectable amount of gluten contents (Figure 1a,c). 

When the conventional heat treatment was applied (Figure 4b), low significant differences among the content of reactive SH groups of heated and untreated gluten were noticed (*p* < 0.05). Besides, the content of total SH groups decreased after the heat treatment at 50 and 100 °C from 1.2 to 0.3 µmol/g, respectively (*p* < 0.05). Microwave heating of gluten proteins at 50 °C with input power 200 W (Figure 4c) appeared to lead to an increase in the reactive SH groups due to unfolding of proteins but with accompanying aggregation, causing a decrease in the total SH content, and probably an increase in disulfide bonds. During the experiment performed at fixed input power of 200 W, the different time treatment caused various temperatures. It can be emphasized that longer exposure time caused higher temperature (100 °C) due to which gluten proteins aggregates were formed, i.e., gliadin molecules were polymerized and cross-linked with glutenins. At the same time, the oxidation of free SH groups occurred, S-S bonds were formed and consequently cross-linking of gliadin aggregates with glutenins were evident. Obviously, MWT caused a higher effect in the range from 60 to 90 °C on both reactive and total SH groups’ content in comparison to heat treatment, particularly at 90 °C, indicating different mechanism of gluten protein denaturation by heat and microwave. It is evident that microwave power and temperatures caused certain changes on protein molecules that seemed to have some relation to the gluten protein unfolding and aggregation. The stated assumptions are in full accordance with the literature data, which analyzed effect of thermal treatment on the gluten structure [44,48]. However, it is very important to state that there is no data in the literature on the use of temperature-controlled microwave reactor, which is the main purpose and benefit of this research.

Theoretically, the major role of SH groups lies in the determination and stabilization of the three-dimensional structure of proteins, thus, the change in SH content by heating represents a first indication that some of the fundamental structural changes in gluten protein functionality exists. It is universally known that glutenin subunits develop ordered fibrous macromolecular polymers with intermolecular disulfide linkages, while gliadins only form intramolecular disulfide linkages [51]. Thus, it can be concluded that the influence of microwave-controlled heat treatment is reflected in terms of the protein native state unfolding and making the gluten protein chains more accessible to Alcalase penetration which is reflected in an enhanced enzymatic hydrolysis reaction. Additionally, these results suggest that MWT at 200 W input power might induce enzymatic hydrolysis cleavage of an existing protein aggregate and the SH groups might take a part in this phenomenon by reducing S-S bonds, which are responsible for maintaining aggregates’ structure.

**Figure 5:**
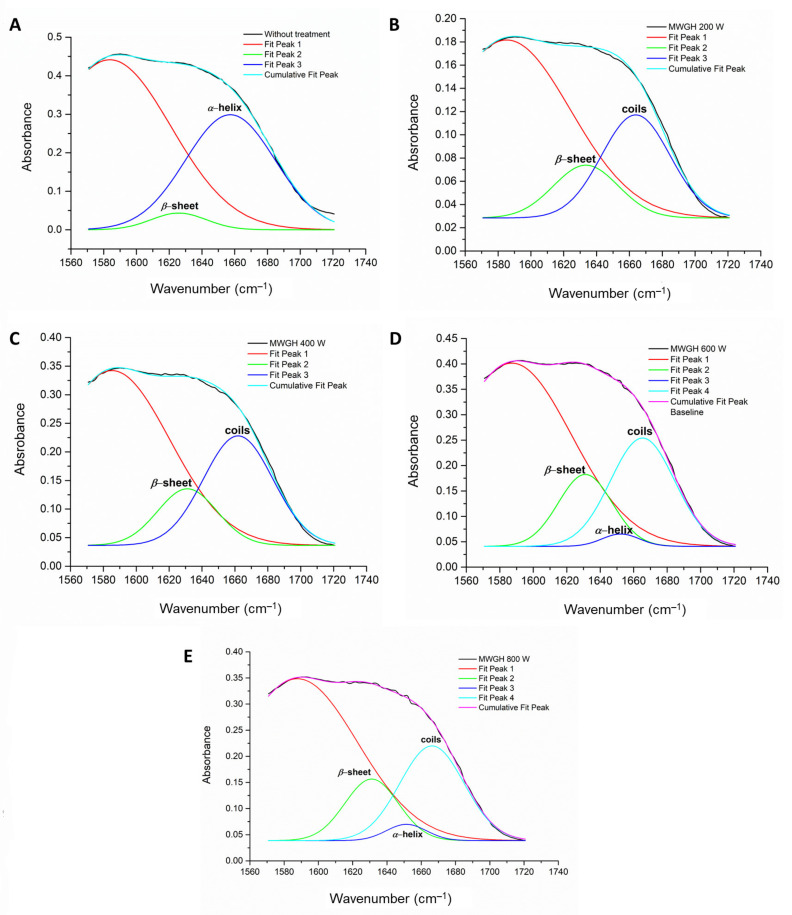
Peak deconvolution of Amide I band in the FTIR spectra of hydrolysates of gluten proteins (**A**) without pretreatment and microwave pretreated at powers of (**B**) 200 W, (**C**) 400 W, (**D**) 600 W and (**E**) 800 W using Peak and Baseline functions (baseline subtracting, deconvolution, second derivate and Gaussian fitting mode) in the OriginPro Lab 9.0.

**Figure 6:**
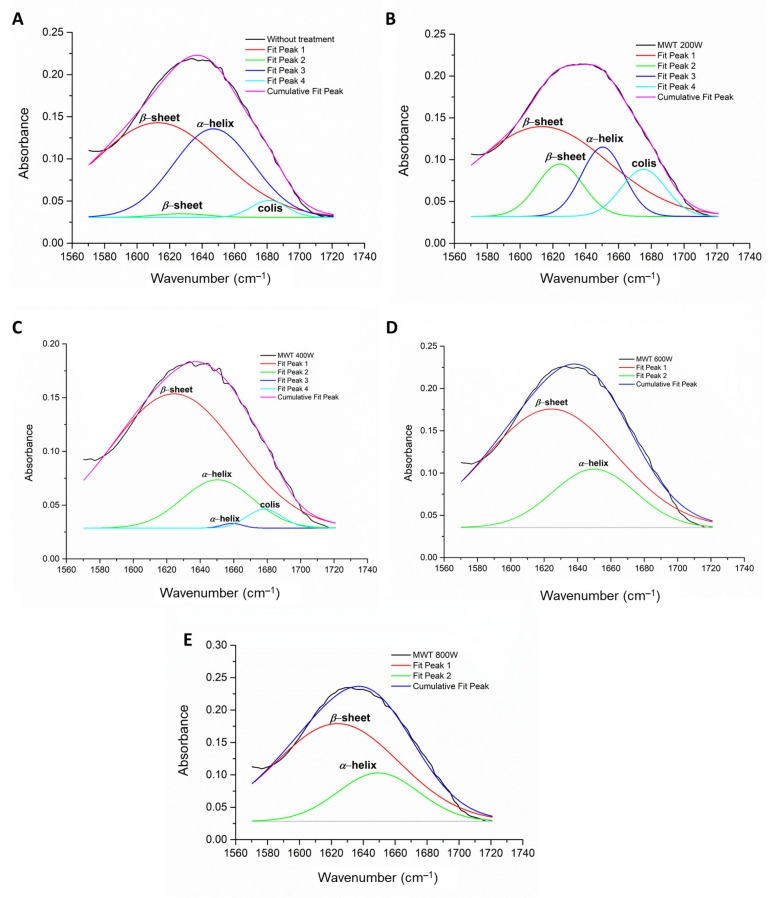
Peak deconvolution of Amide I band in the FTIR spectra of gluten proteins (**A**) without pretreatment and microwave pretreated at powers of (**B**) 200 W, (**C**) 400 W, (**D**) 600 W and (**E**) 800 W using Peak and Baseline functions (baseline subtracting, deconvolution, second derivate and Gaussian fitting mode) in the OriginPro Lab 9.0.

The effects of microwave-controlled treatment on the gluten secondary structure of the solid microwave-treated and hydrolyzed samples were analyzed by FTIR (Figure S1). The deconvolution of the Amide I band (Figure 5 and Figure 6) of control untreated and microwave treated glutens, as well as gluten hydrolysates, enabled the analysis of the main secondary structure elements, namely the relative intensities of the extended *β*-sheet (1623–1641 cm^−1^), the intermolecular *β*-sheet (1612 cm^−1^), the *α*-helix (1648–1657 cm^−1^) and the coils (2 to 7 *α*-helices coiled together; 1662–1686 cm^−1^). The relative intensities of the gluten main secondary structure elements are summarized in Table 1.

The secondary structure of gluten samples and hydrolysate samples significantly changed when the sample was pretreated with microwave-controlled irradiation at different powers of 200–800 W, in comparison to samples without treatment. When the microwave power of 200 W was applied, the total *α*-helix decreased, whereas the *β*-sheet and coils significantly increased with the time. With the increase in the applied power from 200 to 800 W, the contribution of *α*-helix was significantly augmented. It can be emphasized that the *β*-sheet (intermolecular) and *α*-helix of the gluten protein molecules, without treatment and after microwave pre-treatment, were found in the interior of polypeptide chains, and *β*-sheet (extended, i.e., *β*-turn) were formed because of the reversal of polypeptide chains just after microwave pre-treatment with 400, 600 and 800 W. The promotion of *β*-helix and extended *β*-sheet existed due to contribution of the microwave irradiation in reactor system to the formation of disulfide bonds, which is in accordance with the previously discussed results in the Figure 3a, or iso-peptide bonds [44,52].

On the other hand, the intensity of bands assignment in hydrolysates of microwave pretreated gluten clearly demonstrated that microwave irradiation had induced facilitated hydrolysis by Alcalase. The decrease in the relative abundance of *α*-helix secondary structures suggested a different arrangement of the polypeptide bonds in the hydrolysates of microwave treated glutens, especially after application of microwave powers at 200 and 400 W. Considering this observation, it can be stated that combination of microwave pretreatment and Alcalase hydrolysis promoted intermolecular reactions, so that to increase the structural reorganization of *α*-helix to coils (28.0% and 31.3% at 200 and 400 W, respectively), and augmented the relative abundance of side chains (58.3% at 200 W). Extended *β*-sheet structure was significantly changed after synergistic effect of microwave treatment and Alcalase hydrolysis, suggested that partial hydrolyzed polypeptide chains formed more stable structures, which may be crucial for further techno-functional properties and allergenicity.

Comprehensively, the main absorption bands of proteins appear due to the presence of Amide I band stretching vibrations of C=O group (≈1645 cm^−1^) and Amide II band in plane N-H bending vibrations (≈1545 cm^−1^) [37,38]. Noticeable change in the spectra was observed in the Amide I and II bands region (≈1700–1200 cm^−1^) of the hydrolysates. Untreated wheat gluten showed a sharp band at ≈1633 cm^−1^ (Supplementary Figure S2, which changes its shape as shown in Figure S3), where all hydrolysates showed a band at ≈1590 cm^−1^, which was supposedly a result of structural changes in the secondary structure of the protein.

The absorption peaks in the wavelength range of 3100–3500 cm^−1^ (Amide A), which is attributed to N-H and O-H vibrations of the hydroxyl groups, have changed the shape and the intensity after microwave pretreatment due to the interaction of OH vibrations of water molecules and N-H stretching caused by enzymatic hydrolysis of gluten proteins. The applied microwave treatment led to changes in the intensity of tensile vibrations of C–H_2_, C–H and ꞊C–H bonds at wavelength 1450, 1456, 2933 and 3060 cm^−1^, which can be related to the hydrophobic interactions and conformational changes of molecules caused by the action of electromagnetic waves. Compared to the control hydrolysates, the more pronounced absorption bands of all obtained gluten hydrolysates were observed at the wavelength of 1300–1700 cm^−1^ corresponding to the Amide I and Amide II regions, which means that there were structural changes within the amide bond. In particular, the peak losses at 1516 cm^−1^ were observed compared to the control gluten hydrolysis without pre-treatment. Additionally, an increase in peak intensity at 1651 cm^−1^ was observed in all gluten hydrolysates obtained from microwave-pretreated proteins, compared to the control. This increase in peak intensity was a confirmation of the existence of a certain proportion of the *α*-coil within the hydrolysis structure. It is necessary to point out that reduced peak intensity was observed within the Amid VI region at 560 cm^−1^ for all gluten hydrolysates, and the changes or/modifications for gluten microwave pre-treated molecules, compared to the control untreated gluten. Vibrations of the S–S bonds are detected at this wavelength, thus it can be confirmed that there was a change in the number of disulfide connections after microwave treatments at various power. This observation confirmed the previously stated claim that microwave-controlled pre-treatment had an effect on increasing/decreasing the content of sulfhydryl groups in the gluten protein molecules.

**Figure 7:**
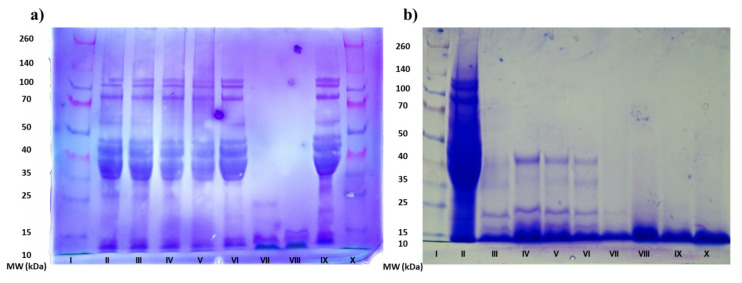
SDS-PAGE profiles of Gluten, MWT gluten at different microwave power (200–800 W), CGH and MWGH. (**a**) Band: I—protein standard, II—MWT 200 W, III—MWT 400 W, IV—MWT 600 W, V—MWT 800 W, VI—Gluten, VII—CHG, VIII—MWGH, IX—Gluten, X—protein standard; (**b**) Band: I—protein standard, II—Gluten, III—CGH, IV—CGH (at 45 min), V—CGH (at 90 min), VI—CGH (at 135 min), VII—MWGH, VIII—MWGH (at 45 min), IX—MWGH (at 90 min), X—MWGH (at 135 min).

Additionally, the denaturation and aggregation of protein molecules after microwave treatment were analyzed by using the denatured SDS-PAGE electrophoresis (Figure 7). The SDS-PAGE results (Figure 7a) clearly demonstrated that both glutenin and gliadin fractions from the MWT (200–800 W) gluten proteins did not present any significant difference in the presented electrophoretic profiles and also in comparison with the control gluten, including any increase or decline in the number of protein bands. The demonstrated migration pattern observed for all the MWT gluten samples pointed out that the primary structure of the gluten proteins was not modified, so microwave treatment with controlled heat effect did not have influence on the gluten protein hydrolysis even at high temperature. Results are in a good accordance with the available literature data, which generally stated that electromagnetic irradiation caused by microwave oven cannot modify the primary structure of proteins, since the energy of the chemical bonds is superior to the quantum energy of the microwave [44,53].

SDS-PAGE electrophoresis showed a different profile between two tested hydrolysates (Figure 7b). The complete absence of protein fractions with molecular weights greater than 45 kDa after enzymatic hydrolysis was observed in both samples. However, difference in detected protein fractions occurred for the molecular weights lesser than 50 kDa. For the 200 W microwave pretreated gluten hydrolysate, a complete absence of gliadin and glutenin fractions in the range from 25–50 kDa was observed. This absence of fractions corresponding to LMW-GS and ω1,2-gliadins (32–39 and 39–44 kDa, respectively), γ- and *α*/*β*-gliadins (31–35 and 28–35 kDa, respectively) can be ascribed to the effect of microwave treatment applied on gliadin and glutenin fractions. Since CGH did not undergo any heat treatment prior to enzymatic hydrolysis temperature equilibration at 60 °C, it was clear that fast and short application of microwave energy of 200 W was sufficient to induce changes in the gluten protein fractions, making them more available to the enzyme. Singh and MacRitchie [54] found that the polymerization of glutenins occurred at temperatures below 100 °C, while gliadins polymerized at higher temperatures. The competitive ELISA test indicated that microwave treatment of wheat gluten reduced detectable gliadin content to a significant level, which is similar with findings of Lamacchia et al. [48].

### 3.4. Beneficial Effect of the Enzymatic Hydrolysis on Antioxidant and Functional Properties

Given the fact that gluten proteins are responsible for the formation of the network and structure of many food products, after examining the influence of controlled microwave pretreatment on the structural changes, the availability of peptide bonds to the biocatalyst and the amount of allergenic toxic epitopes, it was important to examine whether the synergistic effect of enzyme hydrolysis and microwave pretreatment affected techno-functional properties and antioxidant activities of the hydrolysates. Thus, the emulsifying activity index (*EAI*) and emulsion stability (*ESI*) have been tested for untreated gluten, CGH and MWGH, and results are presented in Table 2.

The results indicated that depending on the processing and microwave pretreatment conditions, enzymatic hydrolysis can improve both antioxidant and functional properties of gluten. The most significant improvement (*p* < 0.05) in *EAI* was achieved for the MWGH after 135 min of hydrolysis (50.66 ± 3.10 m^2^/g) compared to raw wheat gluten (11.63 ± 1.10 m^2^/g) or CGH after 90 min (36.19 ± 0.08). However, extensive hydrolysis reduced the *EAI* value, which is in accordance with the literature data for completely hydrolyzed protein samples [26,55]. Smaller peptides appeared to form weaker coats around the oil droplets, thus yielding lower *EAI* values. The extensive hydrolysis of the wheat gluten samples, which generated smaller peptides, resulted in 25.63 ± 2.85 m^2^/g and 22.05 ± 3.60 m^2^/g for the CGH and MWGH, respectively. On the other hand, *ESI* increased with the degree of hydrolysis compared to untreated gluten and the greatest improvement in stability was achieved for the completely hydrolyzed CGH of 3.30 ± 0.31 h. It can be assumed that Alcalase has hydrolyzed the gluten proteins in a way that showed great specificity towards peptide bonds rich in hydrophobic amino acids. Due to the greater exposure of hydrophobic amino acid residues on the surface of molecules, greater ability of gluten hydrolysates to form stable emulsion systems also manifested.

The basic prerequisites for a food protein to be a good foam agent is the ability to be rapidly adsorbed during bubbling process at the air-water interface and undergo a rapid conformational change and rearrangement of functional groups at the interface, and to have the ability to form cohesive viscoelastic film using intermolecular interactions. Thus, prepared hydrolysates, which originated from the microwave-modified and non-modified gluten proteins, were also analyzed on the ability to form cohesive and stable foam (Table 2). It is evident that hydrolysates prepared using the non-extensive action of an endo-protease, Alcalase, i.e., with hydrolysates with lower *DH*, especially after ~45 min of proteolysis where *DH* of ~17% was achieved, possessed the highest foam capacity. This applied to both the hydrolysates obtained from untreated gluten (60.67 ± 1.41%) and the hydrolysates obtained from microwave pre-treated proteins (60.93 ± 3.26%). Further continuation of hydrolysis led to a statistically significant (*p* < 0.05) reduction in the ability to form foam in both cases. The microwave treatment led to different Alcalase activity and substrate specificity, causing a slightly better foam capacity of the hydrolysates. Therefore, the microwave pretreatment had greater effect on the partial aggregation of gluten proteins that were subsequently hydrolyzed by Alcalase, and during hydrolysis, lower molecular weight peptides were formed, with low net charge and high surface hydrophobicity and as such, were ideal to form foam of higher capacity and stability than gluten proteins without treatment. In other words, due to changes in the structure of gluten proteins (*SH* groups content and structural changes referred by the FTIR analysis) caused by microwave pretreatment and subsequently under the influence of Alcalase, they take such a conformation that they allowed the reduction in the boundary stress between the aqueous and air phases. The formed foams showed high stability during 30 min: for CGH stability was measured as 55.05 ± 4.43% and for MWGH was 53.32 ± 0.54%. Yalcin et al. [56] reported that the emulsifying and foaming ability and stability values of microwave-heated gluten samples were slightly greater than those of the control sample, which is in accordance with the results of this research. An identical conclusion was reached in an earlier study examining the effect of Alcalase on the hydrolysis of traditional treated gluten proteins on functional properties [55]. However, it was difficult to discuss microwave-induced effects because microwave devices with precise control of the microwave power and temperature were not used in most cases.

In terms of the potential health benefits of gluten hydrolysates, the antioxidant ability was measured. Therefore, the free-radical scavenging activity determined by using the ABTS˙^+^ radical showed significant improvement in the gluten hydrolysates compared to untreated wheat gluten proteins (Table 2). However, no significant difference was achieved between MWGH and CGH without pretreatment. MWGH had 70.29 ± 1.09% of ABTS activity. All of the samples taken after 45, 90 and 135 min of hydrolysis showed no significant difference in ABTS activity among the tested samples. It can be concluded that after 45 min of hydrolysis, a plateau of free-radical scavenging activity was achieved. In comparison to raw wheat gluten, ABTS IC_50_ values for all of the hydrolysate samples were significantly reduced. Since no significant difference was achieved by microwave pretreatment, it can be concluded that it did not improve the production of more active free-radical scavenging peptides. Metal-ion chelating activities of hydrolysates were also improved by enzymatic hydrolysis in comparison to raw wheat gluten. MICA activity of raw wheat gluten could not be determined. However, in this case, the hydrolysates showed significant difference between MWGH and CGH. The highest MICA of 96.00 ± 0.11% was achieved for the MWGH, revealing that the microwave treatment improved the metal-ion chelating activity of gluten hydrolysates.

## 4. Conclusions

It appeared that microwave specific non-thermal effects had significant influence on the gluten structure and gluten allergenicity, enhancing its hydrolysis and, in combination with the enzymatic hydrolysis, ultimately yielded protein hydrolysates with enhanced free-radical scavenging and metal-ion chelating activity. The combination of enzyme hydrolysis and microwave reactor pretreatment (200 W, 100 °C, 1 min) seemed to be an efficient procedure for gluten content reduction, resulting in almost 10-fold reduction in immunoreactive epitopes with R5 competitive ELISA. Future studies on immunoreactivity of different soluble and insoluble gluten fractions, particularly clinical trials, are required to additionally understand mechanism of inactivation of gluten toxic epitopes by this combined procedure.

## Figures and Tables

**Table 1:** Secondary structure band assignments in the gluten protein samples pretreated with microwaves and in their hydrolysates.

Secondary Structure	Band Assignment in Gluten Microwave Treated Samples				
	Without Treatment	200 W	400 W	600 W	800 W
*α*-helix	38.9%	16.6%	17.3%	25.6%	26.2%
random	n.d.	n.d.	0.49%	n.d.	n.d.
*β*-sheet (extended)	1.03%	13.7%	78.8%	74.4%	73.8%
*β*-sheet (intermolecular)	56.8%	57.7%	n.d.	n.d.	n.d.
coils	3.36%	11.9%	3.04%	n.d.	n.d.
**Secondary Structure**	**Band Assignment in Hydrolysates of Microwave Pretreated Gluten**				
	**Without Treatment**	**200 W**	**400 W**	**600 W**	**800 W**
*α*-helix	41.9%	n.d.	n.d.	1.77%	2.80%
*β*-sheet (extended)	3.77%	13.7%	13.8%	14.8%	14.1%
coils	n.d.	28.0%	31.3%	26.6%	26.0%
side chain	54.3%	58.3%	54.9%	56.8%	57.2%

n.d.—not determined.

**Table 2:** ABTS (%), MICA (%), ABTS IC_50_ (mg_protein_/mL), MICA IC_50_ (mg_protein_/mL), *EAI* (m^2^/g) and *ESI* (h), *FC* (%) and *FS* (%) values for untreated gluten, control gluten hydrolysate (CGH) and microwave pretreated gluten hydrolysate (MWGH).

Sample	ABTS, %	MICA, %	ABTS IC_50_, (mg/mL)	MICA IC_50_, (mg/mL)	*EAI*, m^2^/g	*ESI*, h	*FC*, %	*FS*, %
Gluten	7.52 ± 0.22 ^b^	nd*	63.12 ± 20.85 ^b^	nd*	11.63 ± 1.10 ^f^	0.33 ± 0.04 ^e^	50.93 ± 6.55 ^ab^	48.91 ± 5.13 ^a^
CGH (at 45 min)	66.86 ± 0.88 ^a^	91.24 ± 0.51 ^c^	1.10 ± 0.17 ^a^	1.07 ± 0.00 ^ab^	41.02 ± 2.96 ^b^	1.64 ± 0.85 ^bc^	60.67 ± 1.41 ^a^	55.05 ± 4.43 ^a^
CGH (at 90 min)	69.27 ± 0.66 ^a^	93.44 ± 0.71 ^b^	1.22 ± 0.02 ^a^	0.84 ± 0.05 ^cd^	36.19 ± 0.08 ^b^	1.22 ± 0.47 ^cde^	54.04 ± 0.48 ^ab^	41.27 ± 4.49 ^a^
CGH (at 135 min)	67.45 ± 0.15 ^a^	94.24 ± 0.05 ^ab^	1.11 ± 0.19 ^a^	0.81 ± 0.01 ^cd^	29.29 ± 0.80 ^c^	2.51 ± 0.46 ^ab^	53.09 ± 0.51 ^ab^	38.37 ± 1.64 ^a^
CGH	69.93 ± 1.02 ^a^	94.57 ± 0.09 ^b^	1.08 ± 0.15 ^a^	0.74 ± 0.03 ^d^	25.63 ± 2.85 ^ce^	3.30 ± 0.31 ^a^	47.06 ± 9.31 ^ab^	11.11 ± 7.86 ^b^
MWGH (at 45 min)	66.50 ± 0.51 ^a^	90.12 ± 0.40 ^c^	1.06 ± 0.11 ^a^	1.20 ± 0.09 ^a^	27.59 ± 1.12 ^cd^	3.06 ± 0.42 ^a^	60.93 ± 3.26 ^a^	53.32 ± 0.54 ^a^
MWGH (at 90 min)	69.56 ± 0.51 ^a^	94.75 ± 0.05 ^ab^	1.04 ± 0.14 ^a^	0.84 ± 0.01 ^cd^	27.50 ± 0.95 ^cd^	1.32 ± 0.11 ^be^	56.47 ± 1.29 ^ab^	42.76 ± 8.73 ^a^
MWGH (at 135 min)	68.69 ± 0.51 ^a^	94.80 ± 0.47 ^ab^	0.96 ± 0.06 ^a^	0.92 ± 0.01 ^bc^	50.66 ± 3.10 ^a^	1.10 ± 0.27 ^cde^	59.50 ± 5.05 ^a^	53.7 ± 2.62 ^a^
MWGH	70.29 ± 1.09 ^a^	96.00 ± 0.11 ^a^	1.03 ± 0.17 ^a^	0.75 ± 0.02 ^d^	22.05 ± 3.60 ^de^	1.64 ± 0.30 ^bd^	41.65 ± 1.70 ^b^	10.56 ± 2.74 ^b^

Results are expressed as mean ± standard deviation (*n* = 3). Means with different letters in the same column are significantly different (*p* < 0.05). *nd—could not be determined.

## Data Availability

Not applicable.

## Supplementary Materials

The following are available online at https://www.mdpi.com/article/10.3390/foods10092214/s1, Figure S1: FTIR spectra of the Amide I region (1700–1600 cm^−1^) of the control hydrolysis and microwave pretreated (200–800 W) gluten hydrolysates (Alcalase, pH 8, T 60 °C), Figure S2: FTIR spectra of untreated wheat gluten. Figure S3: FTIR of (a) untreated gluten and MW treated gluten (200–800 W) and (b) gluten, control gluten hydrolysate and MWGH (200–800 W).
