# Supplementary figures and images for: Synergistic Effect of Enzyme Hydrolysis and Microwave Reactor Pretreatment as an Efficient Procedure for Gluten Content Reduction
Source: Foods. 2021 Sep 18;10(9):2214. doi: 10.3390/foods10092214 (PMC8469833; doi:10.3390/foods10092214)

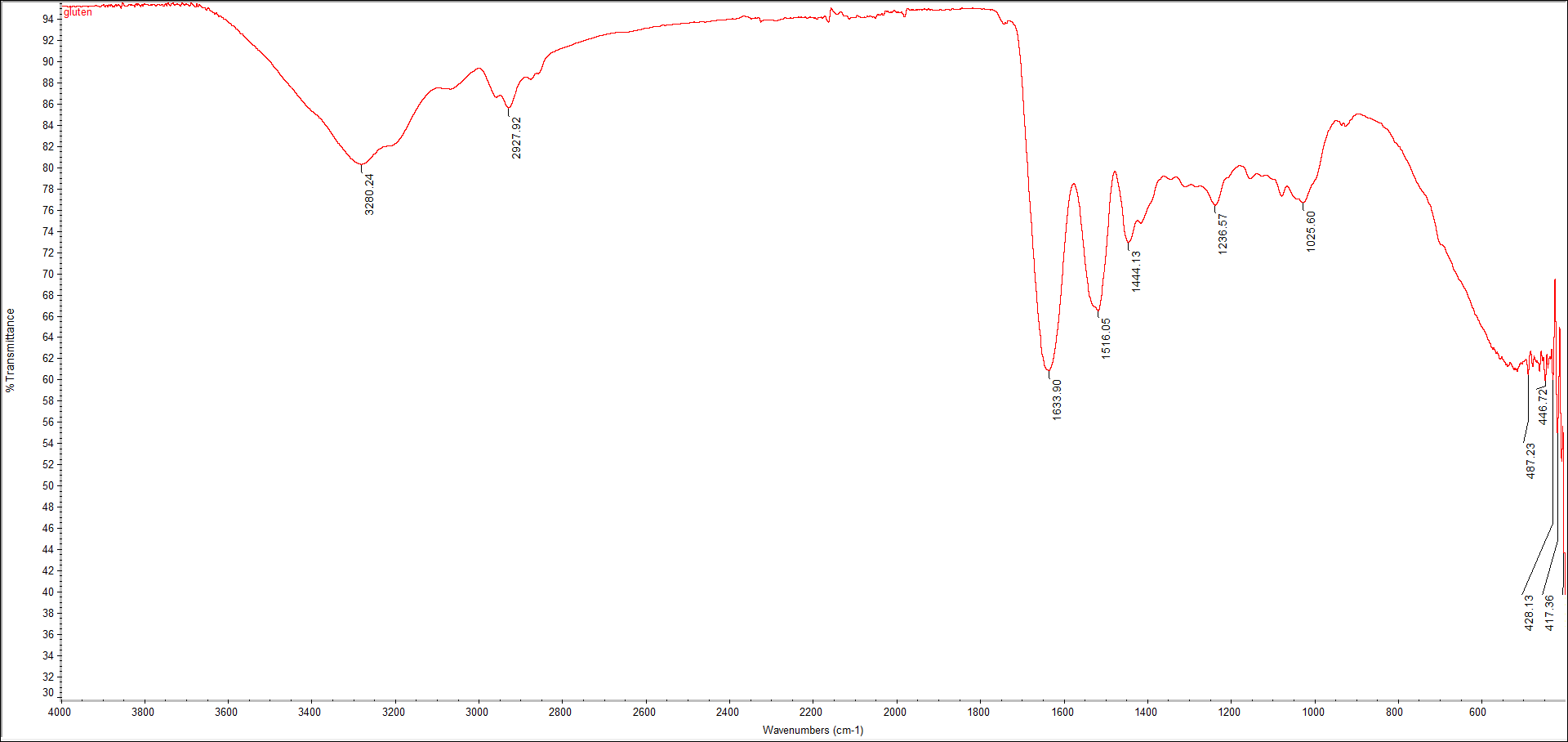

Supplement: Supplementary file 1 [file foods-10-02214-s001.zip › Supplementary figures/Figure S2.TIF]

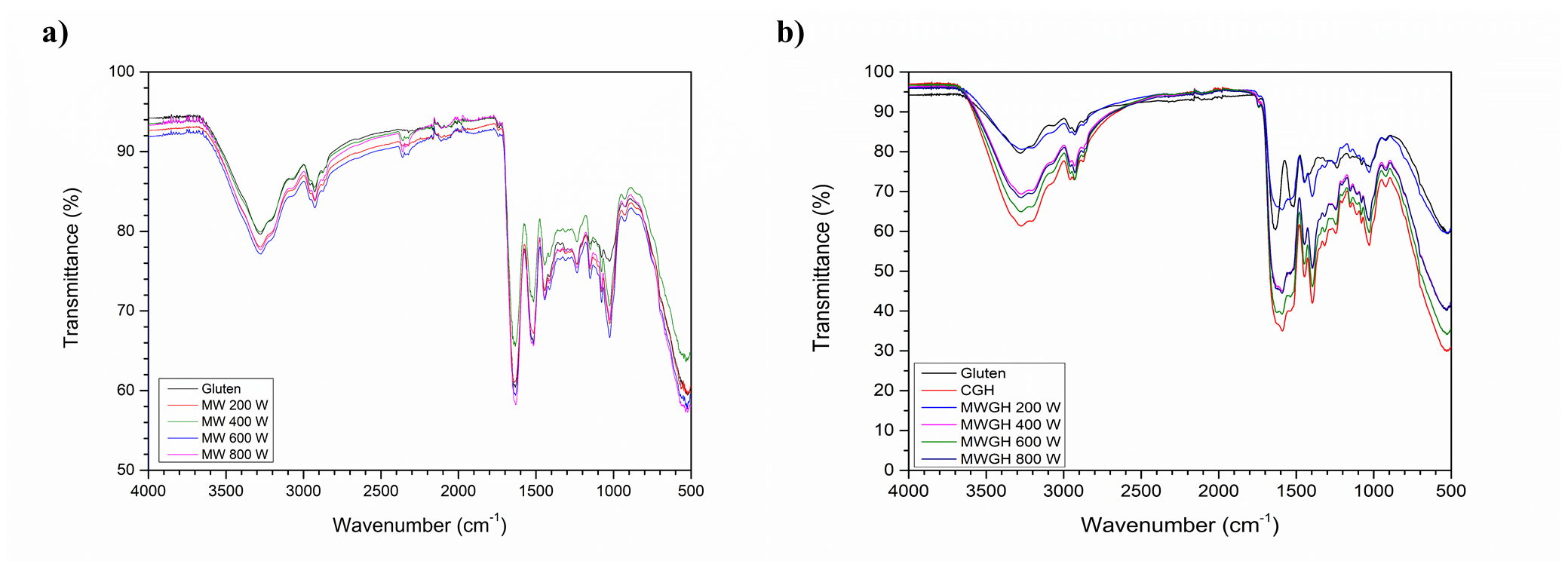

Supplement: Supplementary file 1 [file foods-10-02214-s001.zip › Supplementary figures/Figure S3.tif]
